# NIR Responsive Polymeric Prodrug Micelles ZnPc@P(PEG-CMA-TKGEM) for Combating Gemcitabine Drug Delivery in Anticancer Chemotherapy

**DOI:** 10.3390/ma18174165

**Published:** 2025-09-05

**Authors:** Heng Zhang, Yiping Yang, Shengchao Yang, Yuchang Qin, Xuan Lv, Lin Cui, Wei Jia, Zhiyong Liu

**Affiliations:** 1 Key Laboratory of Green Process for Chemical Engineering, Key Laboratory for Chemical Materials of Xinjiang Uygur Autonomous Region, Engineering Center for Chemical Materials of Xinjiang Bingtuan, School of Chemistry and Chemical Engineering, Shihezi University, Shihezi 832003, China; 2 School of Medical Science, Shihezi University, Shihezi 832003, China

**Keywords:** ROS-responsive, cell imaging agents, photocontrolled drug release, polymer prodrug

## Abstract

The impact of encapsulating gemcitabine (GEM) into nanoparticles on its delivery remains underexplored, with the potential benefits of targeted drug delivery and stimuli-responsive release yet to be fully clarified. Herein, we designed a near-infrared (NIR) light-responsive polymeric nanoparticle, ZnPc@P(PEG-CMA-TKGEM), which integrates reactive oxygen species (ROS) generation and cell imaging capabilities. The self-assembled polymeric micelles exhibit a hydrodynamic size of ~134 nm. Under NIR irradiation, the cumulative drug release rate reaches 51% within 48 h, which is three times higher than that of the non-irradiated control group. In cytotoxicity assays, the cell viability of the NIR-irradiated drug-loaded group is approximately 17%, while that of the NIR-irradiated blank group (without drug loading) remains above 80%. These results confirm that the nanocarriers successfully deliver GEM to target cells and achieve controlled drug release via NIR stimulation.

## 1. Introduction

Gemcitabine (GEM) is a first-line clinical drug for tumor treatment; however, it is easily degraded into inactive metabolites by cytidine deaminase during systemic circulation [1,2,3], leading to low bioavailability and severe side effects such as bone marrow suppression, thrombocytopenia, and leukopenia. Nanoparticle-based drug carriers offer a promising solution for efficient GEM delivery [4]. Nevertheless, GEM is highly hydrophilic, making it difficult to load via conventional adsorption methods. Instead, GEM is typically loaded through prodrug modification [5,6], and achieving precise stimuli-responsive release of such prodrugs remains a critical challenge.

The tumor microenvironment (TME) [7] is characterized by unique features including low pH, mild hyperthermia, hypoxia, high intracellular glutathione (GSH) levels, oxidative stress, and abnormal enzyme expression [8], which have been widely explored for stimuli-responsive prodrug release. However, in vivo, low pH and hyperthermia are not exclusive to tumors but also occur in normal cell microenvironments or inflamed tissues [9]; high GSH levels are also observed in normal cells during oxidative stress responses. Enzyme-responsive systems further suffer from variability in enzyme concentration and tumor heterogeneity. Thus, conventional stimuli-responsive strategies are insufficient to ensure specific prodrug delivery to tumors.

Recent studies have focused on red light and NIR light for stimuli-responsive drug release due to their deep tissue penetration [10]. While red light alone cannot cleave chemical bonds, it can synergize with photochemical agents to generate ROS [11], which can subsequently break various chemical bonds (e.g., selenide bonds, phenylboronic acid esters, and thioketal bonds) to trigger drug release [12,13,14,15,16,17]. ROS-responsive linkers have thus been exploited to develop nanoformulations for antitumor drug delivery, enabling on-demand drug release under ROS stimulation [18,19,20,21]. Another key challenge is enhancing GEM accumulation in tumor tissues via the enhanced permeability and retention (EPR) effect, and polymeric prodrugs have emerged as a viable strategy to address this [22,23]. By conjugating drugs to polymer backbones via responsive linkers [24,25], polymeric prodrugs can achieve high drug-loading stability, making ROS-triggered nano-drug release a promising approach [26].

Despite advances in NIR-responsive drug delivery systems, most reported platforms suffer from two critical limitations:

1. Poor multi-function integration—either lacking real-time imaging capability to monitor drug delivery, or failing to combine ROS-mediated therapy with chemotherapy [10,19].
2. Low release efficiency under NIR stimulation, often due to insufficient ROS generation or unstable stimuli-responsive linkers [27,28]. Additionally, conventional TME-responsive systems (e.g., pH/GSH-triggered) face unavoidable off-target release, as low pH and high GSH are not exclusive to tumors (e.g., inflamed tissues or normal cells under oxidative stress) [9,13].

**Scheme 1:**
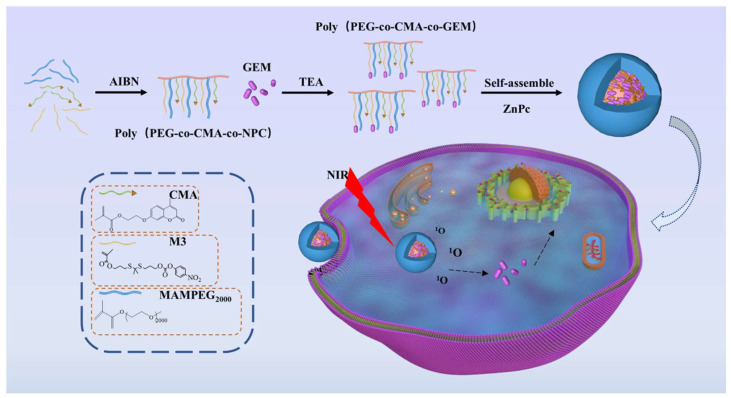
Drug release mechanism of nano-prodrug micelles in tumor cells.

Zinc phthalocyanine (ZnPc) is a photosensitizer with high singlet oxygen quantum yield, widely used in photodynamic therapy (PDT). It generates ROS under NIR irradiation, which can directly act on tumor tissues [27,28,29]. Notably, while previous studies have reported ROS/NIR-responsive drug delivery systems, these systems often lack integration of multiple functions or exhibit limited release efficiency. Herein, we designed a tri-functional NIR-responsive system ZnPc@P(PEG-CMA-TKGEM) that integrates (Scheme 1): (i) ZnPc as a photosensitizer (for ROS generation and red fluorescence imaging); (ii) thioketal (TK) linkers (ROS-specific cleavage, avoiding TME non-specificity); (iii) amphiphilic polymer backbones (PEG for circulation stability, CMA for hydrophobic core formation). This design ensures: (a) protection of GEM from cytidine deaminase degradation via covalent conjugation; (b) spatiotemporal drug release triggered by exogenous NIR (no off-target activation); (c) synergistic chemo-photodynamic therapy (GEM + ROS from ZnPc), which overcomes the low efficacy of single-modal therapy.

## 2. Materials and Methods

### 2.1. Materials and Characterizations

Zinc phthalocyanine (AR, 98%, Shanghai McLean Biochemical Technology Co., Ltd., Shanghai, Chian), 3-mercaptopropionic acid (AR, Titan Technology Co., Ltd., Xi’an, China), dichloromethane (AR, dried over calcium hydride, Tianjin Fuyu Fine Chemical Co., Ltd., Tianjin, China), n-hexane (AR, stored at 2 °C, Tianjin Shengao Chemical Reagent Co., Ltd., Tianjin, China), 2-bromoethanol (AR, Aladdin Biochemical Technology Co., Ltd., Shanghai, China), 7-hydroxy-4-methylcoumarin (AR, Aladdin Biochemical Technology Co., Ltd., Shanghai, China), DMF (AR, dried over calcium hydride), p-nitrophenyl chloroformate (97%, Aladdin Biochemical Technology Co., Ltd., Shanghai, China), methacryloyl chloride (98%, Aladdin Biochemical Technology Co., Ltd., Shanghai, China), lithium aluminum hydride (Aladdin Biochemical Technology Co., Ltd., Shanghai, China), AIBN (recrystallized, Aladdin Biochemical Technology Co., Ltd., Shanghai, China), triethylamine (dried, Aladdin Biochemical Technology Co., Ltd., Shanghai, China), ROS detection kit, double antibody, trypsin digestion solution (Biyuntian Biotechnology Co., Ltd. Shanghai, China), DMEM (Invitrogen), FBS (Zhejiang Tianhang Biotechnology Co., Ltd., Hangzhou, China), gemcitabine (98%, Aladdin Biochemical Technology Co., Ltd., Shanghai, China).

Transmission electron microscope (TEM) images were recorded by a high-resolution transmission electron microscope (HT7700, HITACHI, Tokyo, Japan). The nuclear magnetic resonance hydrogen spectrum was measured by a nuclear magnetic resonance hydrogen spectrum (Bruker DMX-600, Billerica, MA, USA). Zeta potential of samples and hydrodynamic sizes of samples by dynamic light scattering was measured by Zetasizer (NANOPLUS, Micromeritics, Shanghai, China). UV–vis absorption spectra were measured by a UV–vis spectrophotometer (UV-8000S, METASH, Shanghai, China). A Fourier transform infrared spectrometer (FTIR, IRAffinity-1, Shimadzu, Kyoto Prefecture, Japan) was used to evaluate the functional groups in all samples. Confocal microscope images were acquired using a LSM900 microscope from Zeiss, Baden-Württemberg, Germany.

### 2.2. Methods

#### 2.2.1. Synthesis of Polyethylene Glycol Methacrylate (mPEG-MA)

10 g of mPEG2000 and 1.5 g of triethylamine (TEA) were dissolved in 40 mL of dichloromethane (DCM). Under nitrogen atmosphere at 0 °C, a solution of 1.6 g methacryloyl chloride in 10 mL DCM was added dropwise. After reaction at room temperature for 24 h, the ammonium salt byproduct was removed by suction filtration. The organic phase was extracted with saturated sodium chloride solution three times to remove unreacted impurities.

#### 2.2.2. Synthesis of Thioketal (TK) Monomers

A total of 66 g of 3-mercaptopropionic acid and 6.5 g of acetone were added to a flask. An amount of 4 mL of concentrated hydrochloric acid was slowly added dropwise in an ice bath, and the reaction was continued for 2 h. The product was washed with ice water and ice-cold n-hexane, then dried to obtain dicarboxyl-terminated thioketal (M0). Next, 1 g of M0 was dissolved in anhydrous tetrahydrofuran (THF), and 1.6 g of lithium aluminum hydride (LiAlH4) was added in batches under reflux for 1 h. The reaction was quenched by sequential addition of 1.6 g H_2_O, 4.8 g of 10% NaOH aqueous solution, and 4.8 g H_2_O. After cooling, THF was added until no bubbles were generated. The liquid phase was collected by suction filtration and rotary was evaporated to obtain dihydroxyl-terminated thioketal (M1). Finally, 2.69 g of M1 and 1.012 g of TEA were dissolved in 50 mL DCM. A solution of 1.045 g methacryloyl chloride in 20 mL DCM was added dropwise to synthesize a thioketal monomer with one vinyl group and one hydroxyl group (M2). To enable coupling with gemcitabine, 400 mg of M2 and 0.202 g of TEA were dissolved in 5 mL DCM, and a solution of 398 mg p-nitrophenyl chloroformate (NPC) in 5 mL DCM was added dropwise. After reaction completion, the mixture was quenched with 30 mL saturated ammonium chloride, washed with brine, and back-extracted with water. The organic phase was collected, the rotary evaporated, and purified by column chromatography to obtain M3.

#### 2.2.3. Synthesis of 7-(2-Methacryloylethoxy)-4-Methylcoumarin (CMA)

A total of 3 g of 7-hydroxy-4-methylcoumarin, 4.71 g of potassium carbonate, and 30 mL DMF were vigorously stirred for 30 min. A total of 0.142 g of potassium iodide and 2.76 g of 2-bromoethanol were added, and the mixture was heated to 110 °C for 5 h. After cooling, 800 mL of ice water was added, and the mixture was stored at 4 °C for 12 h to precipitate a solid, which was washed with water to obtain crude 7-(2-hydroxyethoxy)-4-methylcoumarin (AMC). Under argon atmosphere at 0 °C, 2.7 g of AMC and 3.3 g of TEA were suspended in 40 mL THF and stirred for 1 h. A solution of 3.5 g methacryloyl chloride in 10 mL THF was added dropwise, and the reaction continued at room temperature for 48 h. The mixture was filtered, the rotary evaporated, precipitated with ice water, suction-filtered, and the filter cake was washed with ice water three times. Recrystallization from ethanol twice yielded white CMA monomers.

#### 2.2.4. Preparation of ZnPc-Loaded Prodrug Polymer Micelles

Polymerization was performed with a mass ratio of hydrophilic to hydrophobic segments of 2:1. AIBN was added as an initiator, and the reaction was carried out at 70 °C for 24 h under argon protection. The product was dialyzed against 40 °C deionized water using a dialysis membrane with a molecular weight cutoff (MWCO) of 7000 Da for 2 days, then at room temperature for 3 h, and freeze-dried to obtain pale yellow polymer P(PEG-CMA-TKNPC). A certain amount of P(PEG-CMA-TKNPC) was added to a flask with 25 mg gemcitabine (GEM), dissolved in DMSO, and 1.5 equivalents of TEA was added. The mixture was stirred at room temperature for 24 h under nitrogen atmosphere and protected from light, then dialyzed for 3 days and freeze-dried to obtain amphiphilic prodrug polymer P(PEG-CMA-TKGEM).

For micelle preparation, 10 mg of P(PEG-CMA-TKGEM) and 0.1/0.2/0.3/0.5/1 mg of ZnPc were dissolved in 2 mL DMSO in the dark. Under stirring at 1500 rpm, 2 mL deionized water was injected via a syringe pump at a rate of 10 μL/min. After stirring for 4 h to stabilize micelles, the solution was dialyzed for 2 days and centrifuged to remove precipitates, yielding ZnPc@P(PEG-CMA-TKGEM) micelle solution. Collect the obtained micellar solution and determine the absorbance of the drug at a wavelength of 274 nm. Then, calculate the drug loading content (DLC) and drug encapsulation efficiency (DLE) using Equations (1) and (2), respectively:

(1) $$DLC(\%)=\frac{M_{1}}{M_{0}}\times 100,$$

(2) $$DLE(\%)=\frac{M_{1}}{M_{0}+M_{2}}\times 100.$$

M_0_ represents the total mass of the added drug (ZnPc); M_1_ represents the mass of the drug encapsulated in the micelles; M_2_ represents the mass of the added carrier (polymer P).

#### 2.2.5. Transmission Electron Microscopy (TEM) Characterization

Take 10 μL of micelle solution (concentration 1 mg/mL) and add it dropwise to a copper mesh (200 mesh, coated with a carbon support film). Let it sit at room temperature for 5 min to allow the micelles to fully adsorb. Then gently blot off any excess liquid with filter paper. Place the copper mesh in a vacuum dryer and dry it for 24 h before measurement.

#### 2.2.6. Dynamic Light Scattering (DLS) and Zeta Potential Characterization

Dilute the micelle solution with ultrapure water to a concentration of 0.1 mg/mL, treat with ultrasound for 5 min (power 300 W) to eliminate aggregation, and measure after standing at room temperature for 30 min. Repeat the measurement three times for each sample, collecting 10 scan data sets each time.

#### 2.2.7. ^1^H NMR Characterization

Weigh 5–10 mg of the sample and transfer it into a clean sample vial. Pipette 0.5–0.6 mL of deuterated solvent into the vial, seal the cap tightly, and agitate gently to ensure complete dissolution of the sample. Using a pipette, carefully transfer the dissolved sample solution into a clean NMR tube, taking care to avoid contact of the solution with the upper walls of the tube. Cap the NMR tube securely, invert it gently 1–2 times, and verify that no precipitation is present. Chemical shifts (δ) are reported in parts per million (ppm), and coupling constants (J) are given in hertz (Hz).

#### 2.2.8. Determination of Critical Micelle Concentration

Empty micelle solutions were diluted to concentrations of 0.5 × 10^−3^ mg/mL, 1.0 × 10^−3^ mg/mL, 0.5 × 10^−2^ mg/mL, 1.0 × 10^−2^ mg/mL, 0.5 × 10^−1^ mg/mL, 1.0 × 10^−1^ mg/mL, and 1.0 mg/mL. For each concentration, 2 mL of sample was mixed with 20 μL of 1.0 × 10^−4^ mg/mL Nile red solution, shaken in the dark for 6 h, and the fluorescence emission spectrum was measured (Cary Eclipse fluorescence spectrophotometer).

#### 2.2.9. Release Behavior of Polymer Prodrugs

ZnPc@P(PEG-CMA-TKGEM) micelles containing ~0.5 mg GEM were added to dialysis bags. The NIR-irradiated group was exposed to NIR light for 1 h before transferring to dialysis bags. Each dialysis bag was placed in a tube containing 15 mL release medium. At predetermined time points, 2 mL of medium was collected and replaced with 2 mL fresh medium. The ultraviolet absorbance of the collected medium at 268 nm was measured to calculate GEM release efficiency.

#### 2.2.10. Detection of Singlet Oxygen (^1^O_2_)

To qualitatively evaluate the generation of singlet oxygen (^1^O_2_), 1,3-diphenylisobenzofuran (DPBF) was employed as a fluorescent probe. For the experimental group, 2 mL of ZnPc@P(PEG-CMA-TKGEM) dispersion (100 μg/mL in DMSO) was uniformly mixed with 1 mL of DPBF solution (20 μg/mL in DMSO). The resulting samples were irradiated with near-infrared (NIR) light for 0 min, 1 min, 2 min, 3 min, and 4 min, respectively. Changes in the UV–visible absorption spectrum at different irradiation time points were monitored using a UV–visible spectrophotometer.

#### 2.2.11. Study on the Generation of Reactive Oxygen Species in Cells Under Illumination

HeLa cells were co-incubated with medium containing prodrug micelles, and cellular uptake was observed via confocal laser scanning microscopy (CLSM) using ZnPc’s red fluorescence. ROS generation after NIR irradiation was detected with DCFH-DA fluorescent probe. HeLa cells (1 × 10^5^ cells/well) were seeded in confocal dishes and cultured until fully adherent. The medium was replaced with micelle-containing medium (200 μg/mL) and incubated for 6 h. After washing with PBS three times, cells were incubated with DCFH-DA probe solution for 30 min, then with diluted probe binding solution for 15 min. Cells were irradiated with NIR light for different durations, washed to remove free probe, fixed with 4% paraformaldehyde, and stained with Hoechst 33342 for 1 h in the dark. After washing, anti-fluorescence quenching mounting medium was added, and images were acquired via CLSM. Red (ZnPc), blue (Hoechst 33342), and green (DCF, oxidized from DCFH) fluorescence were observed under respective excitation wavelengths.

#### 2.2.12. In Vitro Cytotoxicity Experiments

Ten groups of samples were designed: ZnPc@P(PEG-CMA-TKGEM) (with/without NIR), ZnPc@P(PEG-CMA-TKNPC) (with/without NIR), P(PEG-CMA-TKGEM) (with/without NIR), GEM (with/without NIR), and P(PEG-CMA-TKNPC) (with/without NIR). HeLa cells were seeded in 96-well plates at 5000 cells/well and incubated at 37 °C for 24 h until adherent. The medium was replaced with micelle solutions of different concentrations (five concentrations in parallel). The non-irradiated group was incubated for 72 h; the irradiated group was exposed to NIR for 10 min at 24 h, then incubated for another 48 h. CCK-8 solution (100 μL/well) was added, and cell viability was calculated based on absorbance at 450 nm.

## 3. Results and Discussion

### 3.1. Synthesis and Characterization of Polymers

We synthesized a ROS-responsive monomer containing a thioketal linker (M3), along with hydrophobic CMA and hydrophilic MA-mPEG2000 monomers. These monomers were copolymerized via free radical polymerization, followed by conjugation with GEM to yield the ROS-responsive GEM prodrug polymer P(PEG-CMA-TKGEM) (Figure S1, Supporting Information). ^1^H NMR spectroscopy confirmed the successful synthesis of monomers and polymers, as well as the effective conjugation of GEM (Figures S2–S9, Supporting Information). UV-Vis and FTIR analyses further validated the polymer structure: in the UV-Vis spectrum of P(PEG-CMA-TKGEM) (Figure S10), characteristic absorption peaks of CMA (320 nm) and GEM (300–350 nm) were observed, indicating successful conjugation. In the FTIR spectrum (Figure S11), peaks at 1530 cm^−1^ (C=N stretching), 1220 cm^−1^ (C-N stretching), and 1768 cm^−1^ (anhydride C=O stretching) confirmed the presence of GEM-coupled anhydride moieties. Based on ^1^H NMR and standard curve analysis, the GEM content in P(PEG-CMA-TKGEM) was determined to be ~7.28%.

### 3.2. Self-Assembly and Physicochemical Properties of Micelles

**Figure 1:**
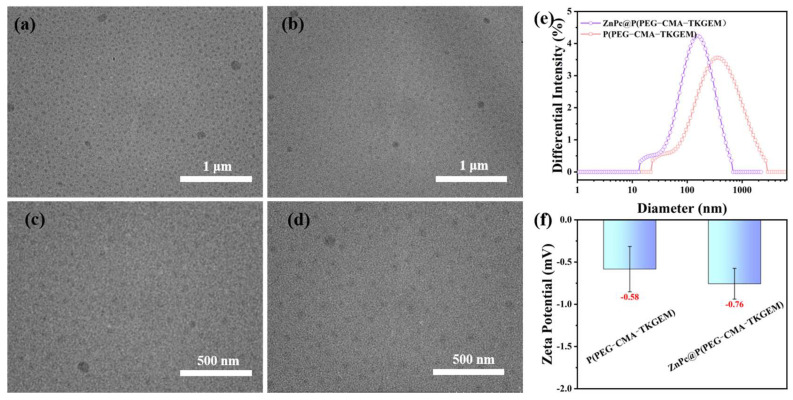
TEM of P(PEG-CMA-TKgem) (**a**,**c**); ZnPc@P(PEG-CMA-TKgem) TEM (**b**,**d**); particle size distribution and zeta-potential (**e**,**f**).

Subsequently, we observed the morphology of nanomicelles at scales of 1 μm and 500 nm using transmission electron microscopy (TEM), as shown in Figure 1. Panels a and c depict the morphologies of micelles formed by P(PEG-CMA-TKGEM) at different scales, while panels b and d illustrate those formed by ZnPc@P(PEG-CMA-TKGEM), respectively. Before and after ZnPc loading, the nanoparticles showed no significant change in size, and both systems self-assembled into spherical nanomicelles. Notably, the nanoparticle size observed via TEM was smaller, which is due to the collapse of nanomicelles during the drying step of sample preparation, whereas the nanoparticle sizer measures the hydrated size of the micellar carriers. Panels e and f of Figure 1 show the particle size distribution and zeta potential results from the nanoparticle sizer, revealing that the nanoparticles exhibited a normal distribution both before and after loading. Specifically, upon loading ZnPc into the micelles, the particle size decreases. The primary reason is the contraction of the hydrophobic core and the reduction in hydration layer thickness. Additionally, enhanced micellar self-assembly stability and structural differences during the drying process may also contribute to this effect. This phenomenon reflects the effective binding between ZnPc and the micelles, while the compact structure and near-electroneutral zeta potential are conducive to improving loading stability and facilitating subsequent applications (e.g., intracellular uptake efficiency in drug delivery).

**Figure 2:**
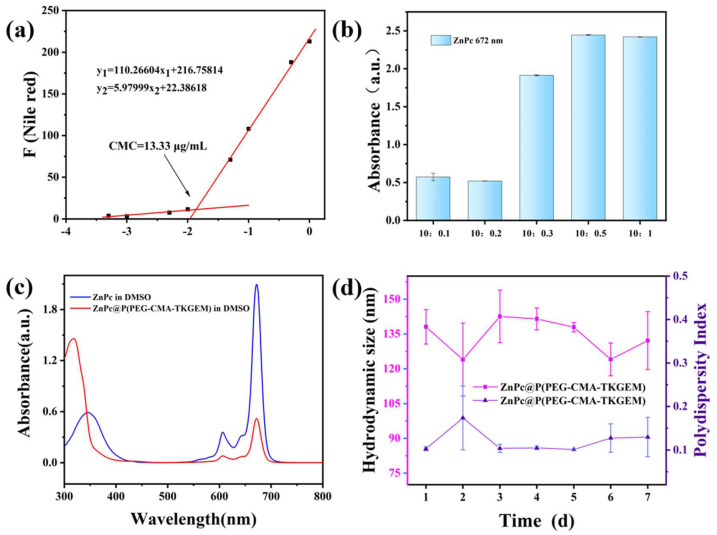
(**a**) The relationship between CMC values and precursor concentration of polymerized GEM; (**b**) the absorbance of ZnPc changes with the feeding ratio; (**c**) UV-Vis spectra of ZnPc and ZnPc@P(PEG-CMA-TkGEM); (**d**) particle size measurement of nano-prodrug micelles within 7 days.

The critical micelle concentration (CMC) was determined using Nile red as a fluorescent probe. Nile red exhibits minimal absorption in aqueous solutions but enhances fluorescence upon solubilization in micellar cores. As shown in Figure 2a, negligible absorption was observed at a micelle concentration of 10^−2^ mg/mL, while solubility increased gradually at 0.5 × 10^−1^ mg/mL. CMC was calculated as the intersection of two linear fits of the absorption data, yielding a value of ~13.33 μg/mL.

To characterize the encapsulation of ZnPc in the micelles, the micelles were first freeze-dried. The lyophilized product was then dissolved in DMSO, and its full absorption spectrum was measured. As shown in Figure 2c, the characteristic absorption peak of ZnPc at 672 nm is clearly observed in the spectrum of the nanocarrier (micelles), confirming the successful encapsulation of ZnPc. This characteristic peak was further used to investigate the maximum loading capacity of the polymer under different polymer-to-ZnPc mass ratios.

To establish a quantitative method for ZnPc, ZnPc solutions with various concentrations were prepared, and their absorbance at 672 nm was determined via UV-Vis spectroscopy. Linear fitting was performed on the concentration-absorbance data (Figure S12), yielding the standard curve equation for ZnPc: A = 0.082C + 0.003 (where A represents absorbance and C represents ZnPc concentration), with a correlation coefficient of R^2^ = 0.9999. The high R^2^ value indicates an excellent linear fitting effect, verifying the reliability of this quantification approach. Subsequently, the drug loading content (DLC) of ZnPc in the micelles prepared at the five different polymer-to-ZnPc mass ratios (as depicted in Figure 2b) was calculated using the aforementioned ZnPc standard curve equation and Equation (1). The results demonstrated that when the polymer-to-ZnPc mass ratio was 10:0.5, the micelles achieved the highest ZnPc loading: the encapsulation efficiency (DLE) reached 59.7%, and the drug loading content (DLC) was 2.9%.

Furthermore, we evaluated the stability of ZnPc@P(PEG-CMA-TKGEM) by monitoring the hydrated size over time (Figure 2d). The diameter of the micelles remained within the range of 120–150 nm over 7 days, with all polydispersity indices (PDIs) below 0.3, indicating good colloidal stability.

### 3.3. NIR-Triggered Micelle Disassembly and Drug Release

**Figure 3:**
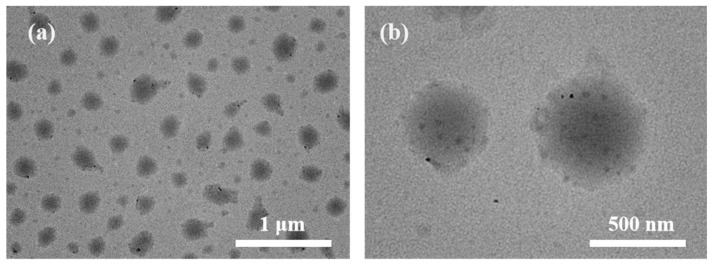
(**a**,**b**) TEM of ZnPc@P(PEG-CMA-TkGEM) after 5 min of NIR irradiation (The image on the right shows a four-fold magnification).

We then investigated the responsive behavior of the micelles under NIR illumination. After NIR irradiation, the diameter of ZnPc@P(PEG-CMA-TKGEM) increased (Figure 3), and a black solid precipitated. This phenomenon was attributed to the generation of ROS upon NIR irradiation: subsequently, the thioketal linkages were cleaved into hydrophilic sulfhydryl groups. The increased hydrophilicity rendered the polymer chains highly unstable, leading to swelling of the inner core and eventual disintegration of the micellar spherical structure, with ZnPc solids precipitating as a result.

**Figure 4:**
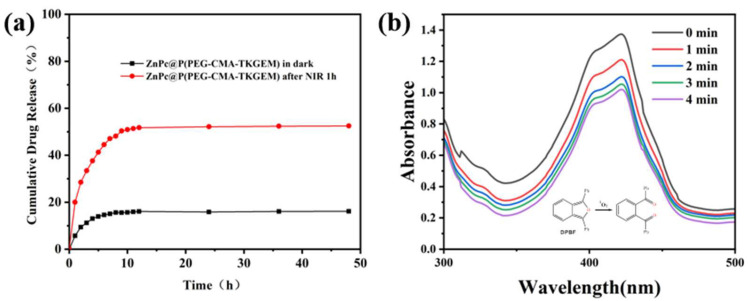
(**a**) Release curves of polymer prodrug micelles under NIR irradiation and darkness for 48 h. The plateau before 100% release is attributed to non-sink conditions, and future studies will optimize the medium volume to address this issue. (**b**) ROS generation ability test of ZnPc@P(PEG-CMA-TkGEM).

NIR irradiation induces ZnPc to generate reactive oxygen species (ROS), which cleave thioketal linkages and trigger the release of GEM. Specifically, ROS-mediated cleavage of thioketal bonds leads to the formation of drug intermediates bearing sulfhydryl groups; subsequently, these sulfhydryl groups attack adjacent carboxyl carbons to form intramolecular rings, accompanied by the release of heterocyclic molecules and free GEM. To validate this proposed mechanism, we evaluated the in vitro release profile (Figure 4a). After 48 h of NIR irradiation, the cumulative release of GEM reached approximately 51%, whereas only 16% of the drug was released in the control group without NIR irradiation. These results indicate that our designed ROS-responsive gemcitabine prodrug polymer micelles achieve effective spatiotemporally controlled responsive release under NIR irradiation. In contrast, in the absence of irradiation, they maintain stable GEM loading and circulatory inertness, thereby effectively preventing premature drug leakage.

Additionally, we employed the 1,3-diphenylisobenzofuran (DPBF) probe to assess the singlet oxygen (^1^O_2_) generation by ZnPc@P(PEG-CMA-TkGEM). The core principle of this experiment is that upon reaction with ^1^O_2_, DPBF undergoes irreversible oxidation, which causes a rapid decrease in its absorption intensity in the UV–visible (UV-Vis) spectrum. As illustrated in Figure 4b, the absorption intensity of DPBF in the ZnPc@P(PEG-CMA-TkGEM) system gradually decreases with prolonged NIR illumination. This result confirms that the material generates a substantial amount of ROS (specifically ^1^O_2_) under NIR irradiation, demonstrating its strong ROS-generating capability. Consequently, this ROS production enables the cleavage of ROS-responsive thioether bonds within the polymer prodrug micelles under these conditions, thereby achieving stimuli-responsive drug release.

### 3.4. Cellular Uptake and ROS Generation

We characterized the endocytic behavior of polymer micelles at the cellular level and observed more clearly the reactive oxygen species (ROS) release behavior of the carriers ZnPc@P(PEG-CMA-TKNPC) and ZnPc@P(PEG-CMA-TKGEM) under different irradiation durations. Physically embedded ZnPc exhibits red fluorescence upon excitation at 630 nm. DCFH-DA itself is non-fluorescent and can freely traverse the cell membrane; once inside the cell, the probe is hydrolyzed by intracellular esterases to generate DCFH, which cannot penetrate the cell membrane. As intracellular ROS levels increase, non-fluorescent DCFH is oxidized to fluorescent DCF, whose fluorescence can be detected under FITC excitation (manifested as green fluorescence).

**Figure 5:**
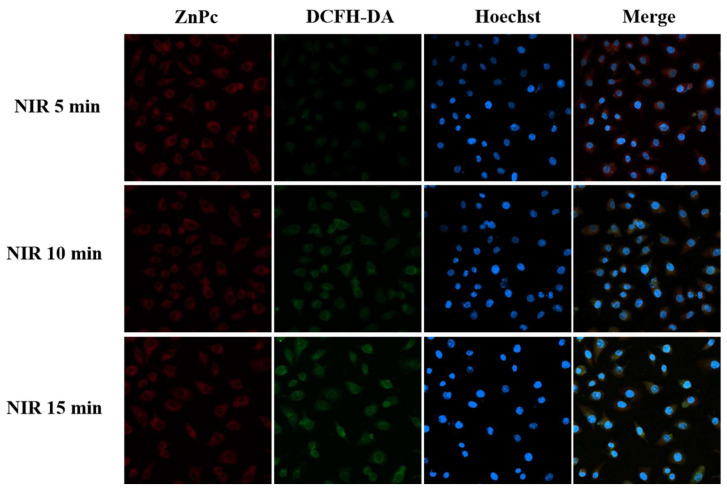
Fluorescence imaging of Hela cells at ZnPc@P(PEG-CMA-TKGEM) irradiated at NIR for 5 min, 10 min and 15 min. Scale bars are 25 µm.

After internalization into cells, ZnPc within ZnPc@P(PEG-CMA-TKNPC) (Figure S13) and ZnPc@P (PEG-CMA-TKGEM) (Figure 5) emitted red fluorescence, allowing visualization of polymer entry. The intensity of red fluorescence correlated with the number of nano-prodrug micelles internalized, with stronger fluorescence indicating higher cellular uptake; microscopy images showed that red fluorescence was predominantly localized in the cytoplasm. This is because ZnPc, released after micelle endocytosis, rarely translocates into the nucleus. Following NIR irradiation, intracellular ROS levels increased over time, leading to a gradual enhancement of green fluorescence. Green fluorescence was distributed throughout the cell but concentrated primarily between the nucleus and cell membrane, with significant overlap with ZnPc localization. In merged images, red fluorescence of ZnPc dominated at short irradiation times; however, as ROS levels increased, green fluorescence gradually intensified and nearly overlapped with red fluorescence. These results demonstrate that intracellular ROS levels increase significantly with prolonged NIR irradiation, directly confirming the carriers’ ability to generate ROS under NIR stimulation.

### 3.5. Cytotoxicity Evaluation

**Figure 6:**
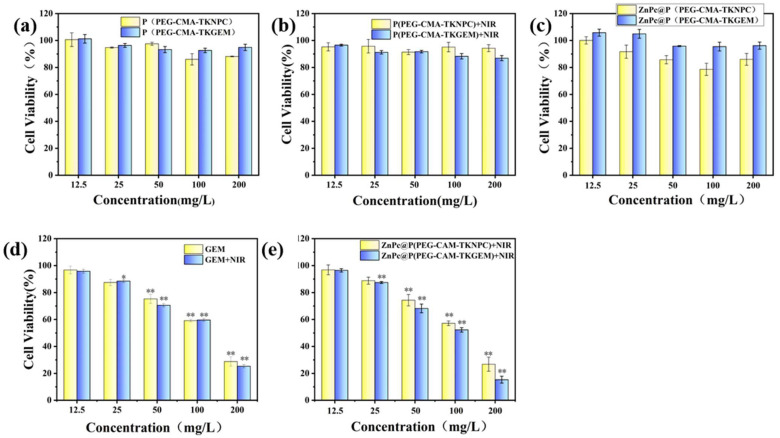
(**a**) Vector toxicity without NIR irradiation and without ZnPc loading; (**b**) Vector toxicity under NIR irradiation without ZnPc loading; (**c**) vector toxicity without NIR irradiation and loaded with ZnPc; (**d**) vector toxicity of GEM with and without NIR; (**e**) vector toxicity under NIR irradiation and loaded with ZnPc. (* *p* < 0.05, ** *p* < 0.01).

For exogenous stimuli-responsive drug delivery systems, carriers should exhibit good biocompatibility in the absence of external stimuli, with no toxic side effects toward cells, while exerting therapeutic effects under stimulation to effectively inhibit tumor cell activity. For the triblock polymers investigated herein—P(PEG-CMA-TKNPC) and P(PEG-CMA-TKGEM)—no cytotoxicity was expected regardless of NIR irradiation. Similarly, ZnPc@P(PEG-CMA-TKGEM) should show low cytotoxicity without irradiation; however, upon NIR illumination, ROS generation and GEM release were anticipated to induce strong cytotoxicity. HeLa cells were coincubated with carriers at varying concentrations under different NIR conditions. The four non-irradiated groups showed that cell viability remained essentially unchanged within 72 h of coincubation, staying above 80% (Figure 6a,c). Furthermore, since TKGEM does not generate toxic degradation products—unlike TKNPC, which releases p-nitrophenol (a toxic byproduct)—the cell viability in the ZnPc@P(PEG-CMA-TKGEM) group is higher than that in the ZnPc@P(PEG-CMA-TKNPC) group in the absence of near-infrared (NIR) irradiation. For micelles lacking ZnPc (Figure 6b), even under NIR irradiation, the ROS released by these micelles alone was far below the lethal threshold, resulting in 72-h cell viability remaining above 80%.

In contrast, the viability of cells treated with ZnPc-loaded micelles decreased gradually under NIR illumination. When the carrier concentration reached 200 μg/mL, the viability of the drug-loaded group dropped to approximately 17% (Figure 6e). The significant difference in cell viability between free GEM and ZnPc@P(PEG-CMA-TKGEM) under NIR irradiation (Figure 6d,e) can be attributed to two key factors. First, the polymeric micelles protect GEM from degradation by cytidine deaminase in circulation, as confirmed by the stable drug retention in non-irradiated groups (Figure 4a). Second, NIR-triggered site-specific release (51% and 16% release) ensures high local GEM concentration at tumor sites, while free GEM is rapidly cleared, leading to lower effective concentration. Additionally, ZnPc-mediated ROS generation may synergistically enhance cytotoxicity by inducing oxidative stress in tumor cells, which is absent in the free GEM group. Notably, in the unloaded group (Figure 6e), cell viability also decreased with increasing carrier concentration; this was attributed to ROS release triggered by NPC molecules, which led to the accumulation of toxic p-nitrophenol in the medium and subsequent reduction in cell viability. These results further confirm that polymer prodrug micelles possess good biocompatibility without exogenous photostimulation. Upon localized NIR irradiation, ROS generation promotes drug release, achieving effective cancer cell eradication. Additionally, prodrug polymers with responsive chemical linkages exhibit lower biotoxicity in the unstimulated state compared to conventional stimuli-responsive encapsulated systems.

## 4. Conclusions

Gemcitabine is a broad-spectrum clinical anticancer drug but suffers from rapid degradation by deaminase, limiting its bioavailability. To address this, we designed ROS-responsive prodrug micelles encapsulating GEM and the photosensitizer ZnPc. Under NIR irradiation, ZnPc generates ROS that cleave thioketal linkers, triggering GEM release. This strategy overcomes insufficient tumor oxidative stress, enhances targeted drug release efficiency, and reduces non-specific leakage and degradation of GEM in the systemic circulation. The developed ZnPc@P(PEG-CMA-TKGEM) system thus represents a promising platform for spatiotemporally controlled anticancer therapy.

## Data Availability

The original contributions presented in this study are included in the article. Further inquiries can be directed to the corresponding authors.

## Supplementary Materials

The following supporting information can be downloaded at: https://www.mdpi.com/article/10.3390/ma18174165/s1, Figure S1: Schematic diagram of monomer synthesis, schematic diagram of polymer and precursor polymer formation; Figure S2: 1H NMR spectra of MA-mPEG2000 (CDCl3); Figure S3: 1H NMR spectra of AMC (DMSO-d6); Figure S4: 1H NMR spectra of CMA (DMSO-d6); Figure S5: 1H NMR spectra of M1 (CDCl3); Figure S6: 1H NMR spectra of M2 (CDCl3); Figure S7: 1H NMR spectra of M3 (CDCl3); Figure S8: 1H NMR spectrum of P(PEG-CMA-TKNPC) (CDCl3); Figure S9: 1H NMR spectra of P(PEG-CMA-TKGEM) (DMSO-d6); Figure S10: UV-Vis spectra of nanoparticles; Figure S11: FT-IR spectra of nanoparticles; Figure S12: Standard graph of ZnPc; Figure S13: Fluorescence imaging of Hela cells at ZnPc@P(PEG-CMA-TKNPC) under NIR irradiation for 5 min, 10 min and 15 min. Scale bars are 25 µm.
