# Supplementary material for: NIR Responsive Polymeric Prodrug Micelles ZnPc@P(PEG-CMA-TKGEM) for Combating Gemcitabine Drug Delivery in Anticancer Chemotherapy
Source: Materials (Basel). 2025 Sep 5;18(17):4165. doi: 10.3390/ma18174165 (PMC12430095; doi:10.3390/ma18174165)
Supplement: Supplementary file 1 [file materials-18-04165-s001.zip › materials-3813446-supplementary.pdf]

S1. Synthesis and Characterization of Monomers and Polymers

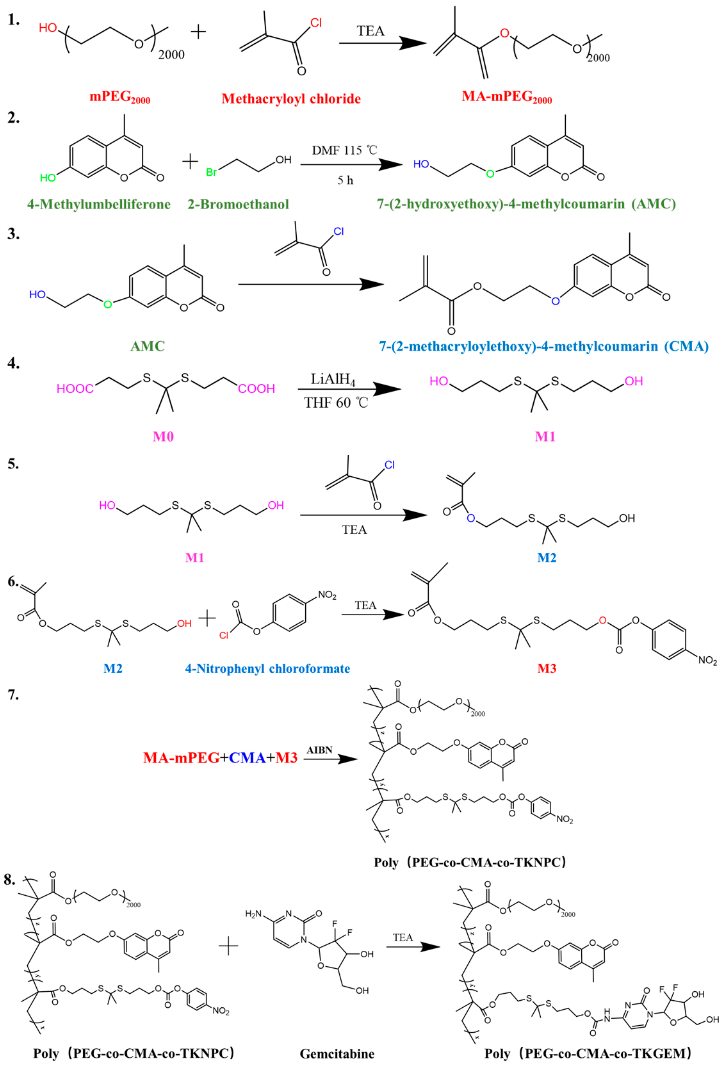

Figure S1. Schematic diagram of monomer synthesis, schematic diagram of polymer and precursor polymer formation.

S1.1 Synthesis of MA-mPEG2000

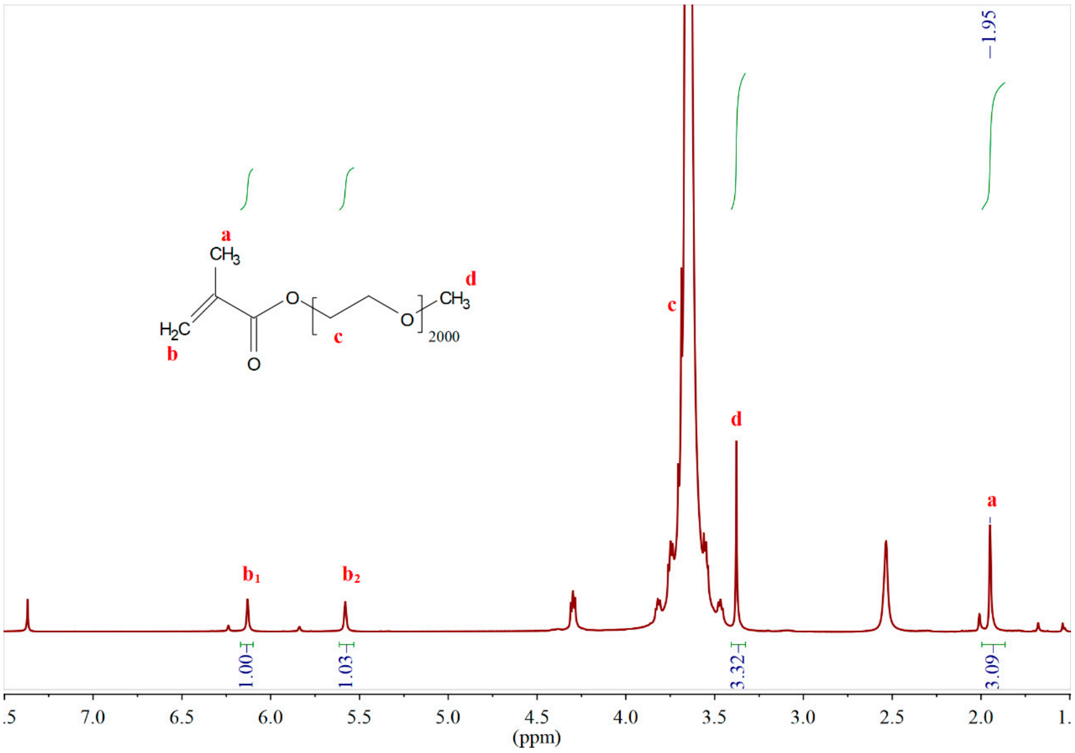

Figure S2. ^1H NMR spectra of MA-mPEG$_{2000}$ (CDCl$_3$).

^1H NMR spectroscopy confirmed the successful synthesis of MA-mPEG2000 (Figure S2). Integration of the spectrum showed a proton ratio of ~1:3 between the vinyl protons of the methacrylate group and the methyl protons, consistent with the expected structure.

S1.2 Synthesis of CMA (7-(2-Methacryloylethoxy)-4-Methylcoumarin)

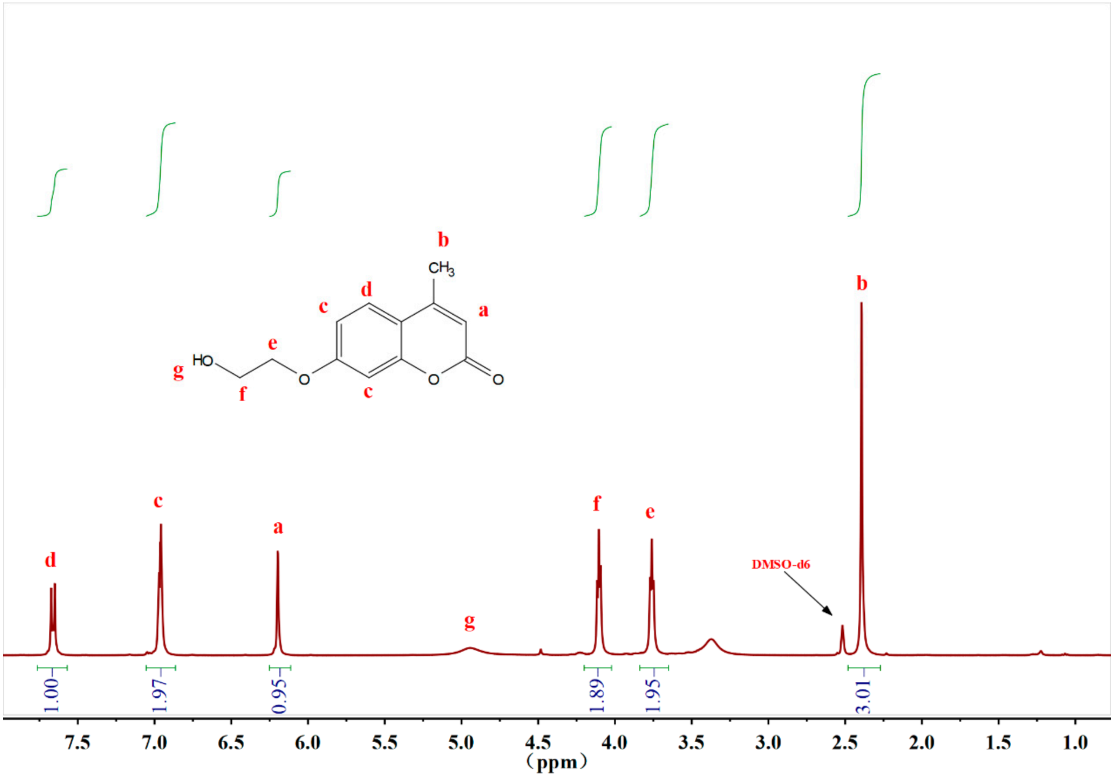

Figure S3. ^1H NMR spectra of AMC (DMSO-d6).

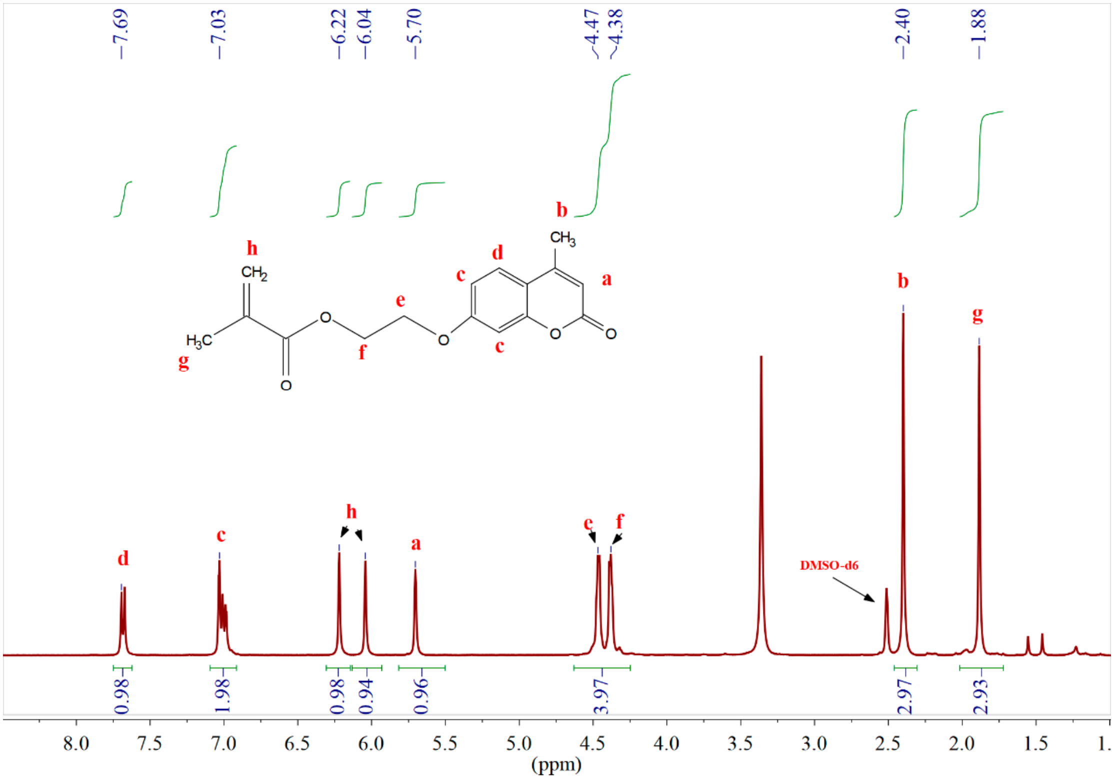

Figure S4. ^{1}H NMR spectra of CMA (DMSO-d6).

7-hydroxy-4-methylcoumarin was reacted with 2-bromoethanol to introduce a reactive hydroxyl group, yielding AMC. As shown in Figure S3, new methylene proton peaks appeared at 3.75 ppm and 4.15 ppm in AMC's ^{1}H NMR spectrum, confirming its synthesis. For CMA (Figure S4), vinyl proton peaks from methacryloyl chloride were observed at 6.04 ppm and 6.22 ppm, and a methyl proton peak at 1.88 ppm, verifying successful introduction of the vinyl group.

S1.3 Synthesis of Thioketal Monomers (M0, M1, M2, M3)

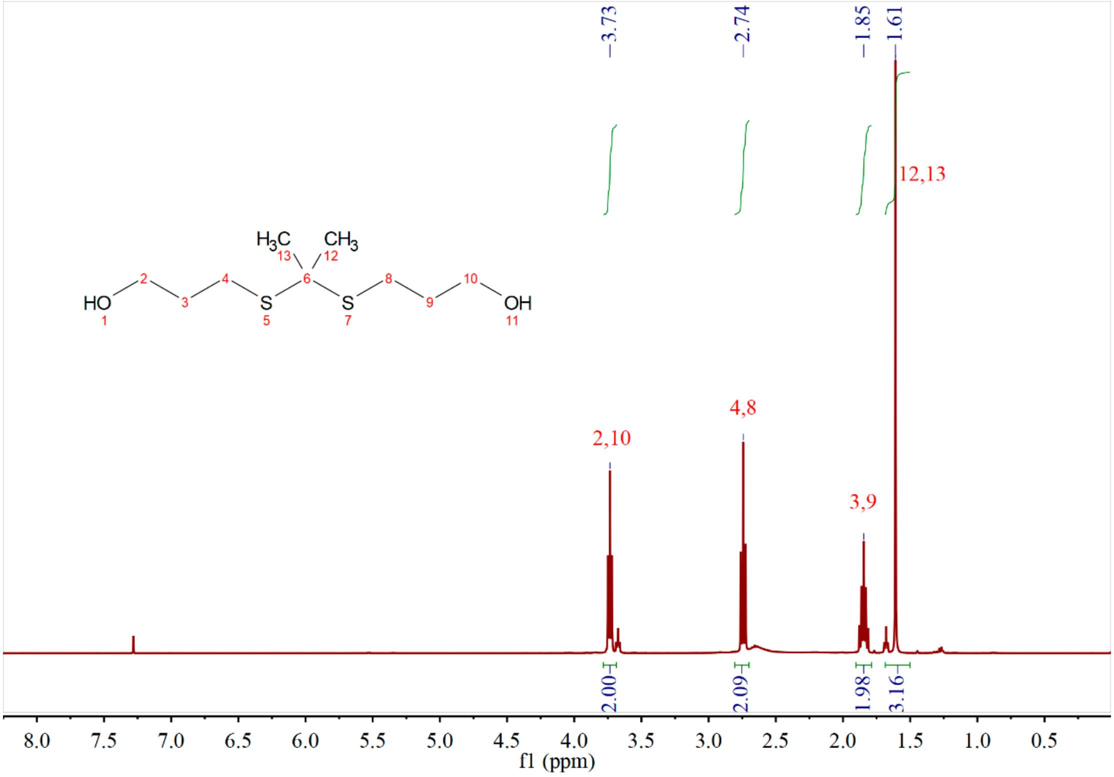

Figure S5. ^{1}H NMR spectra of M1 ($CDCl_3$).

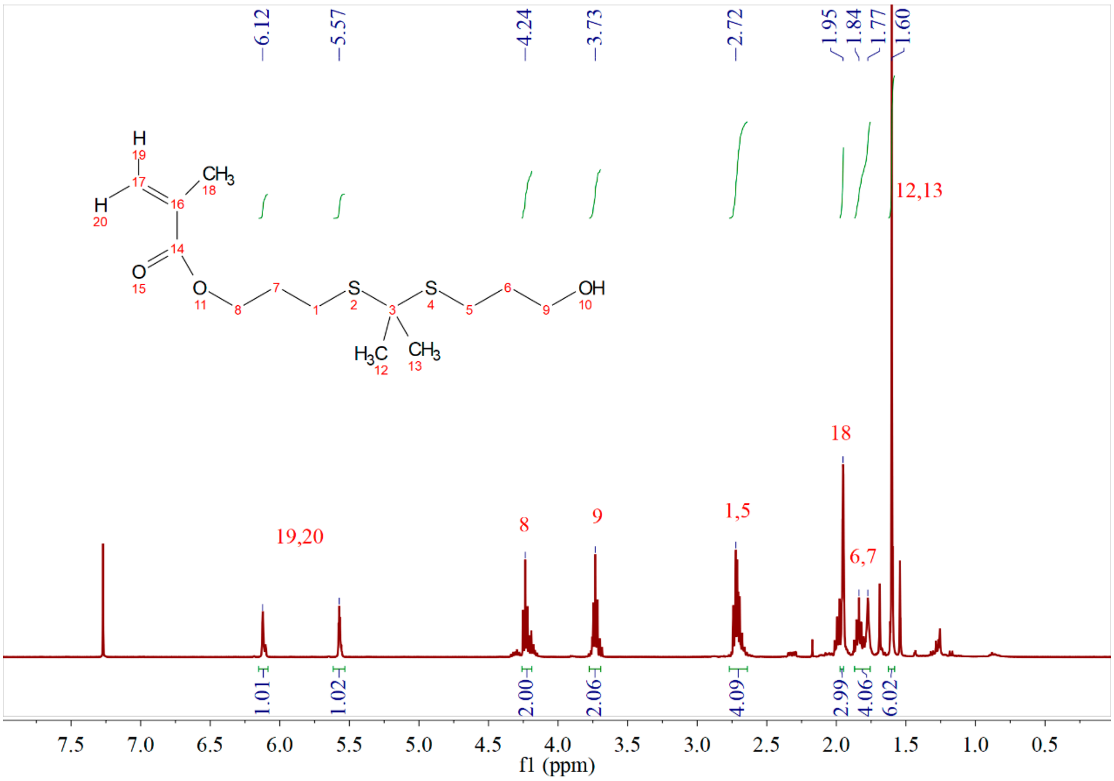

Figure S6. ^{1}H NMR spectra of M2 ($CDCl_3$).

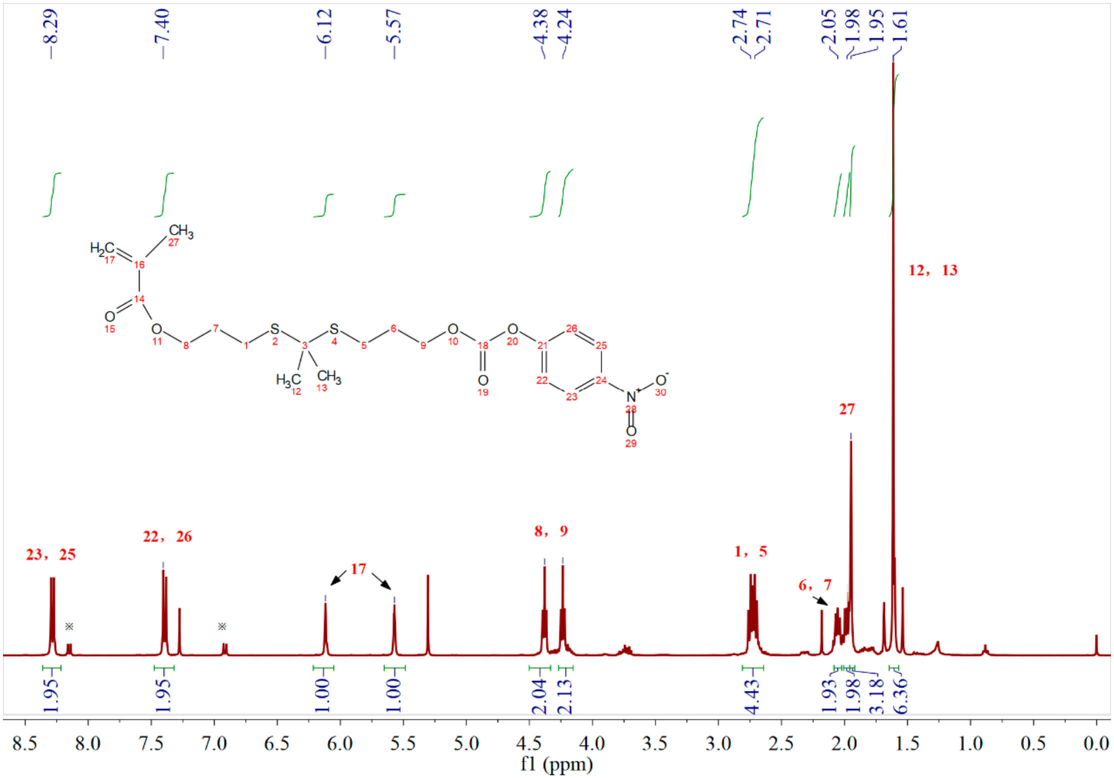

Figure S7. ^1H NMR spectra of M3 ($CDCl_3$).

First, dicarboxyl-terminated thioketal (M0) was synthesized. To enable reaction with methacryloyl chloride, M0 was reduced to dihydroxyl-terminated thioketal (M1, Figure S5). Reaction of M1 with methacryloyl chloride yielded monosubstituted vinyl thioketal monomer M2 (Figure S6), confirmed by vinyl proton peaks at 5.57 ppm and 6.12 ppm, and a methyl proton peak at 1.95 ppm. Due to single-end substitution, the asymmetric structure resulted in a 2:3:6 integral ratio of methylene, vinyl methyl, and thioketal methyl protons at ~1.6 ppm. For M3 synthesis (Figure S7), reaction of M2 with NPC introduced aromatic groups, as evidenced by benzene ring proton peaks at 7.40 ppm and 8.40 ppm. Minor impurity peaks were attributed to residual p-nitrophenol, which was removed by subsequent dialysis in 40 °C water.

S1.4 Synthesis of P(PEG-CMA-TKNPC) and P(PEG-CMA-TKGEM)

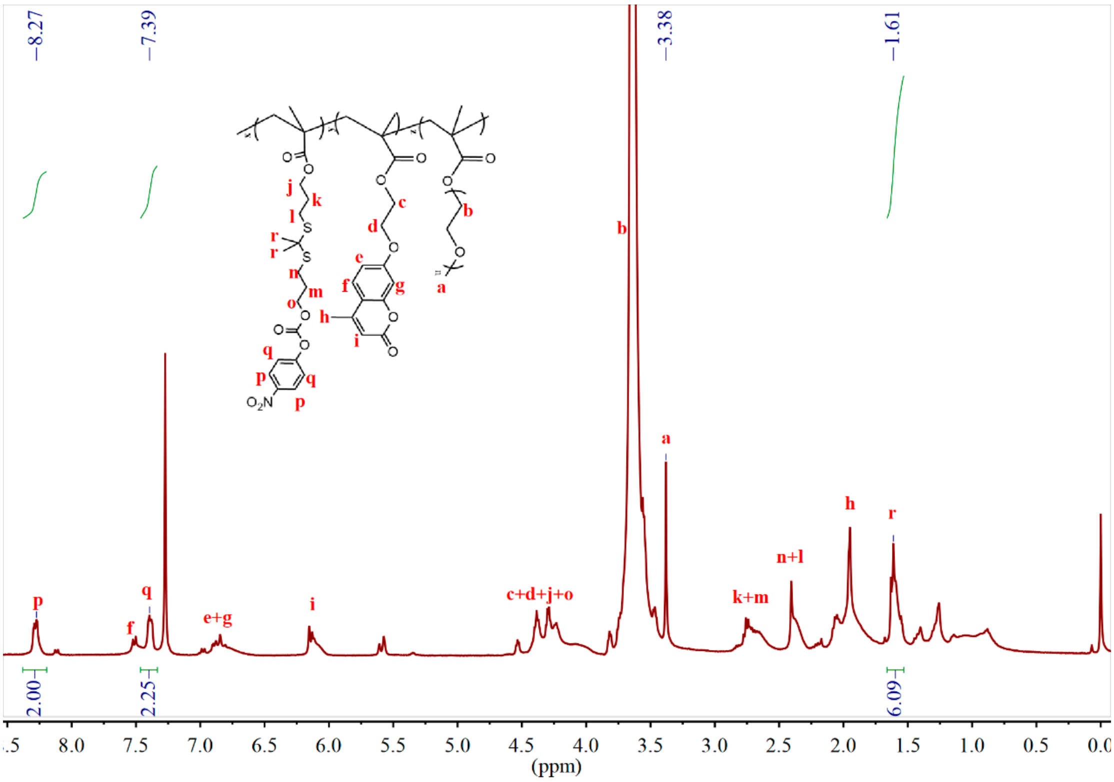

Figure S8. ^{1}H NMR spectrum of P(PEG-CMA-TKNPC) ($CDCl_3$).

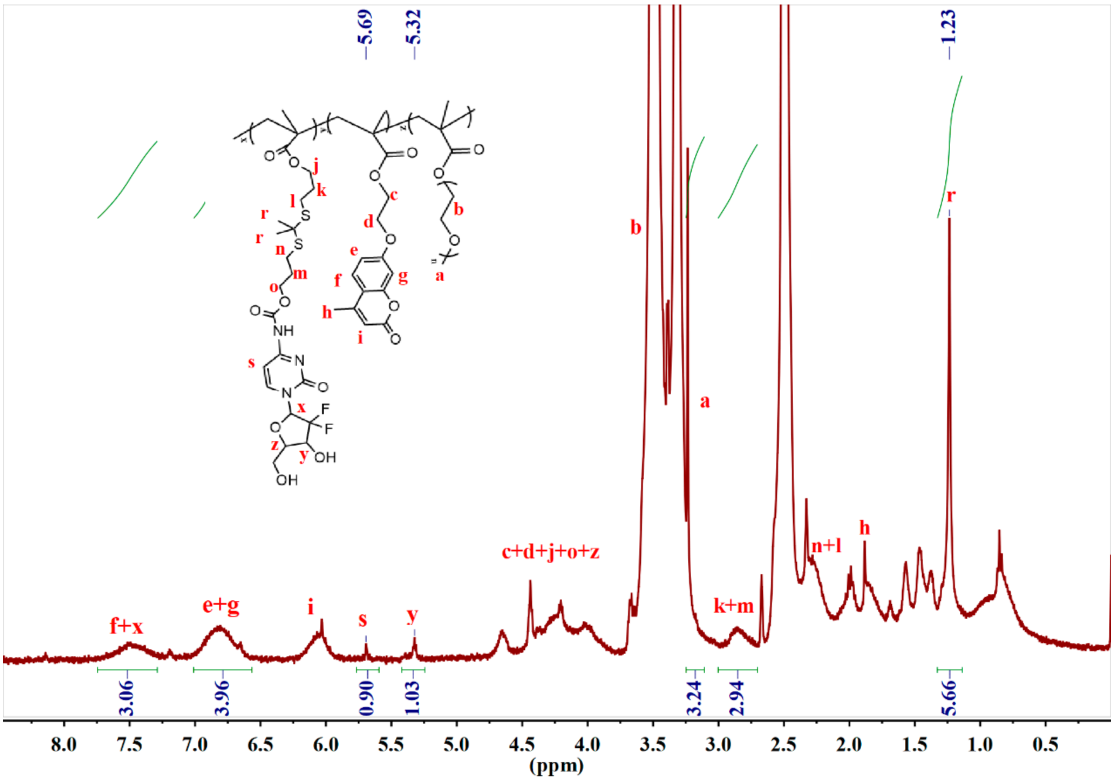

Figure S9. ^{1}H NMR spectra of P(PEG-CMA-TKGEM) (DMSO-d6).

Radical polymerization of M3, MA-mPEG2000, and CMA yielded P(PEG-CMA-TKNPC), confirmed by ^{1}H NMR (Figure S8): PEG methyl proton peak at 3.3 ppm, CMA aromatic proton peaks at 5.5–7.7 ppm, and thioketal methylene/aromatic proton peaks at 2.2–3 ppm, 4.0–4.5 ppm, 7.39 ppm, and 8.27 ppm. Coupling of GEM to P(PEG-CMA-TKNPC) yielded P(PEG-CMA-TKGEM) (Figure S9), as indicated by the disappearance of NPC aromatic peaks (7.39 ppm, 8.27 ppm) and appearance of GEM characteristic peaks at 5.32 ppm and 5.69 ppm. The integral ratio of mPEG:CMA:GEM was ~1:2:1.

S2. Physicochemical Characterization of Micelles

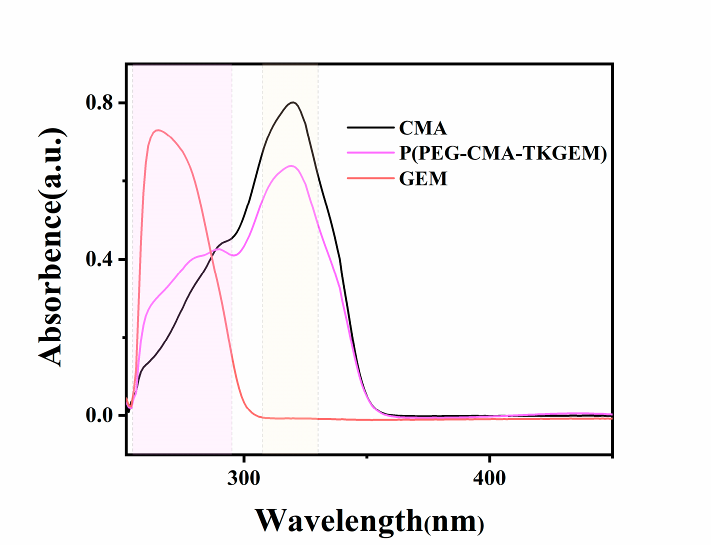

Figure S10. UV-Vis spectra of nanoparticles.

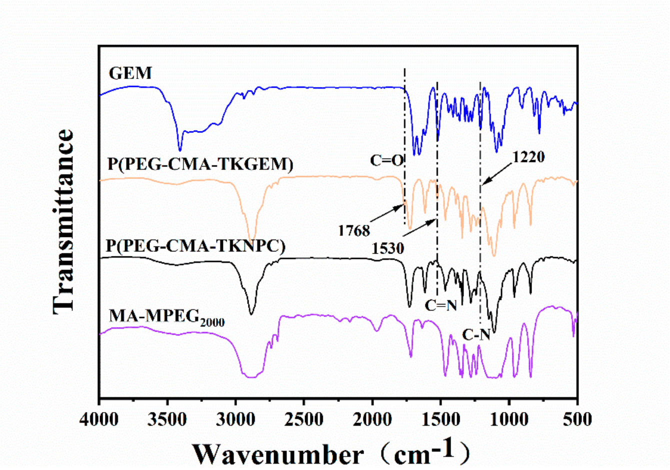

Figure S11. FT-IR spectra of nanoparticles.

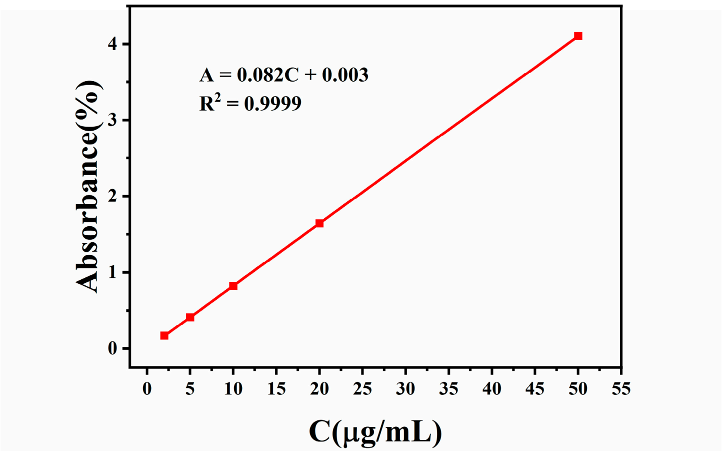

Figure S12. Standard graph of ZnPc.

S3. Cellular Experiments

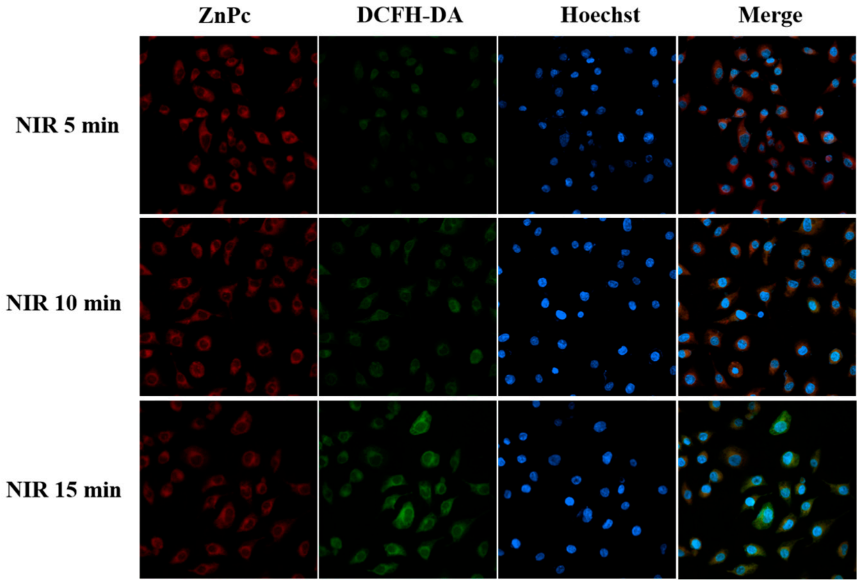

Figure S13. Fluorescence imaging of Hela cells at ZnPc@P (PEG-CMA-TKNPC) under NIR irradiation for 5 min, 10 min and 15 min. Scale bars are 25 μm.
